# Comment on “Are Patients with Polycystic Ovarian Syndrome Ideal Candidates for Oocyte Donation?”

**DOI:** 10.1155/2017/2051620

**Published:** 2017-07-19

**Authors:** Sahni Chetan, Rima Dada

**Affiliations:** Lab for Molecular Reproduction and Genetics, Department of Anatomy, All India Institute of Medical Sciences, New Delhi 110029, India

The article “Are Patients with Polycystic Ovarian Syndrome Ideal Candidates for Oocyte Donation?” by Vaz et al. [1] is important because IVF (in vitro fertilization) offers hope to millions of infertile couples. The demand and use of donated oocytes have increased significantly over the last decade. Although the authors highlight the advantage of using oocytes retrieved from women with PCOS as they have more oocytes retrieved per cycle and require less gonadotrophins, the disadvantage of using such oocytes cannot be ignored.

Though the authors highlight that more oocytes were retrieved (3.23 more oocytes per cycle in women with PCOS), the number of mature oocytes retrieved was similar in both donor groups (PCOS versus Non-PCOS). Also, the fertilization rate, implantation rate, and clinical pregnancy rate were not different. The authors emphasized that this may be due to donor and recipient age and endometrial receptivity. The success of implantation and clinical pregnancy rate also depends on paternal factors, which have been totally ignored in this manuscript. There is strong paternal effect of sperm DNA damage at all stages of embryonic development. These effects have been classified as early (embryo development from day one to four cell stage) and late (day 2 to day 5) paternal effects and implantation effects (Simon et al.) [2]. At the time of fertilization, sperm transmits not only DNA but also mRNA, miRNA, oocyte-activating factor, and centromeres. The integrity of each is a clinical determinant of fertilization and postfertilization events especially cleavage and implantation (Venkatesh et al.) [3]. Kumar et al. [4] highlight the importance of the integrity of sperm DNA and level of sperm transcripts in determining the optimal embryogenesis and live birth rate and also documented that loss of sperm DNA integrity may lead to infertility, pre- and postimplantation losses, congenital malformations, and even cancer. DNA damage is partially corrected and removed by base excision repair (oxoguanine glycosylase-1) in sperm. However, it is dependent on the oocyte for complete removal of DNA lesions. Mutagenic products and mutations accumulate with advanced paternal age due to oxidative stress-induced DNA damage and more premeiotic replication cycles. Thus paternal factors are a critical determinant of embryo developmental potential and competence and may thus determine the implantation and pregnancy rates.

Also, the authors propose that oocytes retrieved from women with PCOS may be used in oocyte donation. However, the altered hormonal milieu in women with PCOS may affect the oocyte epigenome, which is highly dynamic and its effect would be transgenerational. The dysregulated oocyte transcripts would also adversely affect the fertilization, implantation, and pregnancy rates. Oocytes from women with PCOS should be used with caution and the effect of paternal factors, oxidative DNA damage, and dysregulated sperm transmission should be considered while calculating the fertilization, implantation, and pregnancy rate.
